# Dysphagia due to Diffuse Idiopathic Skeletal Hyperostosis

**DOI:** 10.1155/2012/123825

**Published:** 2012-04-12

**Authors:** Masafumi Ohki

**Affiliations:** Department of Otolaryngology, Saitama Medical Center, 1981 Kamoda, Kawagoe-shi, Saitama 350-8550, Japan

## Abstract

Diffuse idiopathic skeletal hyperostosis (DISH) is usually asymptomatic. However, rarely, it causes dysphagia, hoarseness, dyspnea, snoring, stridor, and laryngeal edema. Herein, we present a patient with DISH causing dysphagia. A 70-year-old man presented with a 4-month history of sore throat, dysphagia, and foreign body sensation. Flexible laryngoscopy revealed a leftward-protruding posterior wall in the hypopharynx. Computed tomography and magnetic resonance imaging revealed a bony mass pushing, anteriorly, on the posterior hypopharyngeal wall. Ossification included an osseous bridge involving 5 contiguous vertebral bodies. Dysphagia due to DISH was diagnosed. His symptoms were relieved by conservative therapy using anti-inflammatory drugs. However, if conservative therapy fails and symptoms are severe, surgical treatments must be considered.

## 1. Introduction

Diffuse idiopathic skeletal hyperostosis (DISH) is a disease characterized by massive, noninflammatory ossification with intensive formation of osteophytes affecting ligaments, tendons, and fascia of the anterior part of the spinal column, mostly in the middle and lower thoracic regions [1, 2]. It is called Forestier disease as Forestier was the first to report this disorder [1]. The defining criteria include ossification forming an osseous bridge involving at least 4 contiguous vertebral bodies [2]. Ossification can occur in the cervical region as well. It is mainly an asymptomatic disorder in elderly persons. However, DISH in the cervical region can, in rare cases, cause dysphagia [3]. We herein report a rare case of dysphagia associated with DISH.

## 2. Case

**Figure 1:**
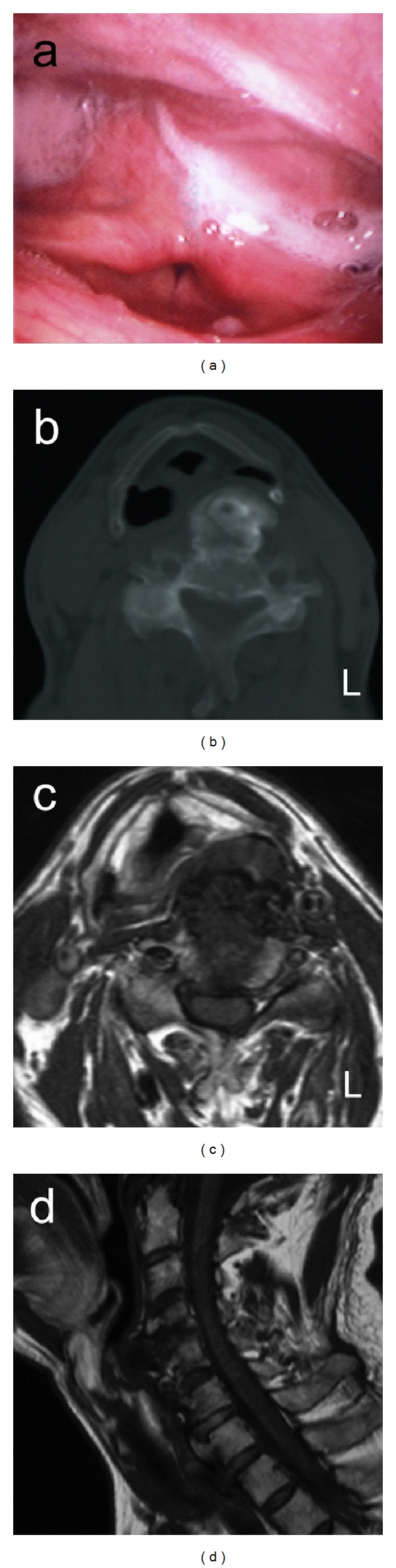
(a) a hard mass protruding in the posterior hypopharyngeal wall. (b) CT scan shows a bony mass pushing the posterior piriform sinus wall anteriorly. (c) Axial T1 weighted-MRI shows a mass pushing the posterior piriform sinus wall anteriorly. (d) sagittal T1 weighted-MRI shows an anterior mass of the cervical spine from C4 to T1.

A 70-year-old man presented with a 4-month history of sore throat, dysphagia, and foreign body sensation. Flexible laryngoscopy revealed a left-sided protrusion in the posterior hypopharyngeal wall (Figure 1(a)). The surface of the protruding wall was smooth and intact, and the protruding mass was hard. Vocal cord mobility was normal. The left piriform sinus of the hypopharynx was narrowed by the protruding wall and saliva pooled within it. Axial computed tomography (CT) (Figure 1(b)) and magnetic resonance imaging (MRI) (Figures 1(c) and 1(d)) of the neck showed a bony mass pushing, anteriorly, the posterior wall of the hypopharynx suggesting dysphagia associated with DISH. One month after staring carbocisteine at 1500 mg and lysozyme hydrochloride 90 mg daily, his symptoms of sore throat and foreign body sensation showed relief. The saliva pooling in the piriform sinus was decreased.

## 3. Discussion

The proposed mechanisms of dysphagia in DISH include mechanical compression, periesopharyngeal inflammation with edema and fibrosis due to chronic irritation, and cricopharyngeal spasm also due to chronic irritation [4–6]. A bolus is blocked at the piriform sinus. Limited mobility of the epiglottis and elevation of the larynx cause dysphagia as well as aspiration. The narrowed airway causes dyspnea [7–10]. Retrocricoid inflammation can induce laryngeal edema [11], dysphonia, vocal cord immobility [12, 13], or stridor [7]. We first applied conservative therapy, that is, anti-inflammatory drugs, muscle relaxants, and so on. Only when symptoms are severe, that is, aphagia, appearance of neurological signs such as compression (myelopathy) [14, 15] or obstruction of the airway [8–10], is surgical treatment indicated. Surgery is performed mainly via the anterior (Smith-Robinson approach) [16, 17] and transpharyngeal approaches [17, 18]. However, we must be aware of potentially severe complications of surgical treatment such as hematoma, Horner syndrome, recurrent nerve palsy, superior laryngeal nerve palsy, and esophageal injury [19]. The anterior approach carries a risk of dysphonia or dysphagia, at rates of 38% and 23%, respectively, for more than 6 months after surgery [16]. Therefore, surgical treatment should be selected with care.

## 4. Conclusion

Dysphagia due to DISH is rare. CT or MRI is useful for diagnosing DISH. Although conservative therapy and surgical treatments are available, surgery carries severe potential risks. Conservative therapy is recommended first and surgical treatment is considered only if conservative therapy fails and symptoms are severe.
